# Characterization of Spectrum, *de novo* Rate and Genotype-Phenotype Correlation of Dominant *GJB2* Mutations in Chinese Hans

**DOI:** 10.1371/journal.pone.0100483

**Published:** 2014-06-19

**Authors:** Xiuhong Pang, Yongchuan Chai, Lianhua Sun, Dongye Chen, Ying Chen, Zhihua Zhang, Hao Wu, Tao Yang

**Affiliations:** 1 Department of Otorhinolaryngology-Head and Neck Surgery, Xinhua Hospital, Shanghai Jiaotong University School of Medicine, Shanghai, China; 2 Ear Institute, Shanghai Jiaotong University, Shanghai, China; Oslo University Hospital, Norway

## Abstract

Dominant mutations in *GJB2* may lead to various degrees of sensorineural hearing impairment and/or hyperproliferative epidermal disorders. So far studies of dominant *GJB2* mutations were mostly limited to case reports of individual patients and families. In this study, we identified 7 families, 11 subjects with dominant *GJB2* mutations by sequencing of *GJB2* in 2168 Chinese Han probands with sensorineural hearing impairment and characterized the associated spectrum, *de novo* rate and genotype-phenotype correlation. We identified p.R75Q, p.R75W and p.R184Q as the most frequent dominant *GJB2* mutations among Chinese Hans, which had a very high *de novo* rate (71% of probands). A majority (10/11) of subjects carrying dominant *GJB2* mutations exhibited palmoplantar keratoderma in addition to hearing impairment. In two families segregated with additional c.235delC or p.V37I mutations of *GJB2*, family members with the compound heterozygous mutations exhibited more severe phenotype than those with single dominant *GJB2* mutation. Our study suggested that the high *de novo* mutation rate gives rise to a significant portion of dominant *GJB2* mutations. The severity of the hearing and epidermal phenotypes associated with dominant *GJB2* mutations may be modified by additional recessive mutations of *GJB2*.

## Introduction

Numerous gap junction proteins, termed as connexins (Cx), are expressed in inner ear and epidermis. Formed by hexameric connexin hemichannels (connexon) between adjacent cells, those gap junctions play an important role in the potassium homeostasis of the inner ear as well as in the keratinocyte growth and differentiation of the epidermis [1].

Mutations in the *GJB2* gene, encoding the gap-junction protein connexin 26 (Cx26), may lead to sensorineural hearing loss (HL) and hyperproliferative epidermal disorder. Recessive *GJB2* mutations are predominantly associated with non-syndromic HL (DFNB1A, MIM220290) and are the most common cause of hereditary HL [2]. In contrast, dominant *GJB2* mutations may cause both non-syndromic (DFNA3A, MIM601544) and syndromic HL [3], [4]. Differing mostly in various types of epidermal disorders, the syndromic HL associated with dominant *GJB2* mutations includes keratitis-ichthyosis-deafness (KID, MIM148210) syndrome, hystrix-like ichthyosis with deafness (HID, MIM602540), palmoplantar keratoderma (PPK, MIM148350) with deafness, Vohwinkel syndrome (MIM124500) and Bart-Pumphrey syndrome (MIM149200). Major epidermal phenotypes associated with dominant *GJB2* mutations include ichthyosis, pseudoainhum, palmoplantar hyperkeratosis, knuckle pads and nail abnormalities [5].

*De novo* mutation occurs in the germline during early embryogenesis or somatically in some overgrowth syndromes [6]. With the advent of whole-exome and whole-genome sequencing approaches, its etiologic contribution to both rare and common genetic diseases has been increasingly recognized [7], [8]. Though on average more deleterious than their inherited counterparts due to less stringent selective pressure [9], *de novo* mutations have nevertheless been reported in some disorders with lesser impact on fitness, including a number of *de novo GJB2* mutations detected in sporadic patients with sensorineural HL and hyperproliferative epidermal disorder [10].

Unlike recessive *GJB2* mutations, for which the mutation spectrum and the genotype-phenotype correlation have been well characterized, previous studies of dominant *GJB2* mutations were mostly limited to case reports of individual patients and families. In the present study, we aimed to systematically characterize the spectrum, *de novo* rate and genotype-phenotype correlation of dominant *GJB2* mutations in Chinese Han patients with syndromic or non-syndromic HL.

## Materials and Methods

### Subject recruitment

A total of 2168 Chinese Han probands with sensorineural HL were recruited from patients seeking genetic testing and counseling in Department of Otolaryngology - Head and Neck Surgery, Xinhua Hospital, Shanghai, China. Among them, 1904 (87.8%) probands had severe or profound HL, while the rest 264 (12.2%) had mild or moderate HL. Additional family members were recruited for those carrying known dominant *GJB2* mutations. All subjects or their parents gave written, informed consent to participate in this study. This study was approved by the Ethics Committee of Xinhua Hospital, Shanghai Jiao tong University School of Medicine.

### Mutation screening of *GJB2*

Genomic DNA was extracted from the whole blood samples using the Blood DNA kit (TIANGEN BIOTECH, Beijing, China). Mutation screening of all exons and flanking splicing sites of *GJB2* was performed by PCR amplification and bidirectional sequencing as previously described [11]. The dominant mutations identified in this study were also screened in 100 ethnically-matched normal hearing controls.

### Auditory and epidermal evaluations

For subjects and family members carrying known dominant *GJB2* mutations, physical examination was performed with special attention to hearing and epidermal abnormalities. Comprehensive auditory evaluations were performed including otoscope examination, tympanometry and pure-tone audiometry (PTA). Hearing threshold was calculated as the average of the hearing levels at 0.5, 1.0, 2.0, 4.0 KHz for the better ear. The severity of hearing impairment was defined as mild (20–40 dB), moderate (41–70 dB), severe (71–95 dB) and profound (>95 dB). Histological examination was performed in skin biopsies of patient C209-1.

### Short tandem repeat analysis

For subjects carrying potential *de novo* mutations of *GJB2*, short tandem repeat (STR) analysis was performed in the affected individuals and their unaffected parents to confirm the parenthood using informative STRs D5S435, D5S629, D5S610, D5S351, D6S1714, D6S1573, D6S1344, D6S469, D3S1597, D3S3611, D3S3601 and D3S3589.

## Results

### Spectrum and phenotypes of dominant *GJB2* mutations

By mutation screening of *GJB2* in 2168 Chinese Han probands with sensorineural HL, we identified 7 (0.32%) probands carrying known dominant *GJB2* mutations p.R75Q (n = 3), p.R75W (n = 2) and p.R184Q (n = 2). Four additional family members carrying p.R75Q (n = 2) and p.R75W (n = 2) mutations were also recruited in this study. All other *GJB2* variants detected in this study were either previously reported recessive mutations or non-pathogenic polymorphisms. None of the three dominant mutations in *GJB2* was seen in 100 ethnically-matched normal hearing controls.

**Figure 1:**
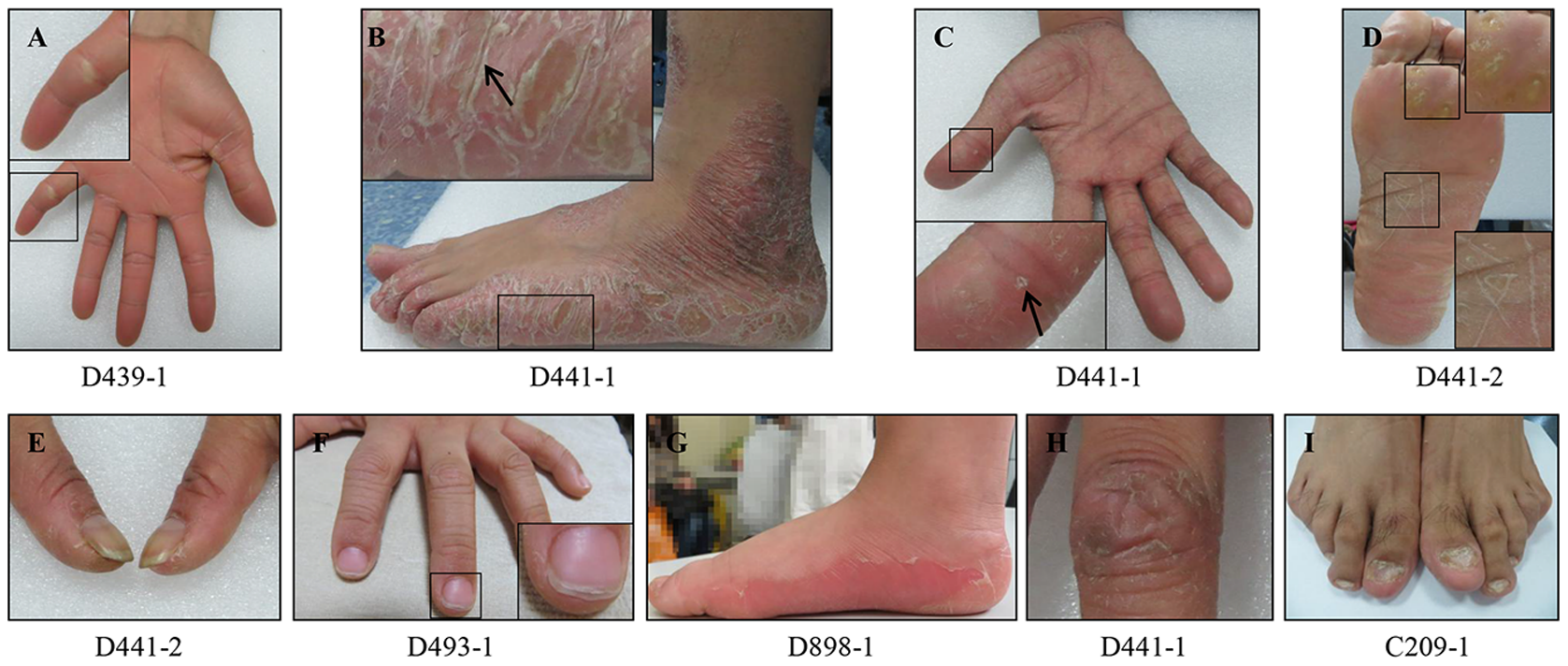
Representative epidermal abnormalities of the subjects carrying dominant *GJB2* mutations. (A) Thickening of the skin and the circular keratotic constriction band (box); (B) Peeling (box) and deep fissures (arrow in the box); (C) Punctiform keratoderma; (D) Callus (the upper box) and striped keratoderma (the lower box); (E) Thickened nails; (F) Brittle nails; (G) Erythema; (H) knuckle pad; (I) spooned nails.

Table 1 summarized the genotypes and phenotypes observed in the 11 subjects carrying dominant *GJB2* mutations. Except for subject D439-2, who had mild HL with delayed-onset, the majority of the subjects (10/11) had congenital, severe-to-profound HL. Ten of the 11 subjects had additional PPK-associated epidermal abnormalities in their hands and feet, including thickening and peeling of the skin, keratoderma, erythema, callus, deep fissures, brittle, thickened or spoon nails, knuckle pads and circular keratotic constriction bands (Figure 1).

**Table 1:** Genotypes and phenotypes of subjects with dominant *GJB2* mutations.

Family	Patient	Relation to the proband	Age (years)	*GJB2* genotype	Hearing impairment	Epidermal abnormalities	Origin of mutation
D439	D439-1*	-	17	p.R75Q/p.V37I	Congenital, profound	Thickening (palms and soles) and peeling (soles) of the skin, keratoderma (palms), erythema (hands and feet), knuckle pads and circular keratotic constriction band (fingers)	Parental inherited
	D439-2	Half sibling	26	p.R75Q/+	Delayed-onset (∼10 years), mild	Minor thickening of the skin (palms only) and keratoderma (palms)	Parental inherited
	D439-4	Mother	49	p.R75Q/p.V37I	Congenital, profound	Thickening (palms and soles) of the skin, keratoderma (palms), knuckle pads and circular keratotic constriction band (fingers), callus (soles)	Unknown
D898	D898-1*	-	3	p.R75Q/+	Congenital, profound	Thickening and peeling of the skin (palms and soles), erythema (hands and feet)	*De novo*
D1057	D1057-1*	-	31	p.R75Q/+	Congenital, severe	Thickening of the skin (palms), keratoderma (palms), knuckle pads (fingers)	*De novo*
D441	D441-1*	-	17	p.R75W/c.235delC	Congenital, profound	Severe peeling (soles) and thickening (palms and soles) of the skin, keratoderma (palms and feet), erythema (hands and feet), knuckle pads (fingers), deep fissures (hand and feet); the epidermal abnormalities were extended to wrists, ankles and the dorsal area of hands and feet	Parental inherited
	D441-2	Mother	44	p.R75W/+	Congenital, profound	Thickening of the skin (palms and soles), keratoderma (feet), erythema (hands and feet), knuckle pads (fingers), callus (soles)	*De novo*
D493	D493-1*	-	5	p.R75W/+	Congenital, profound	Thickening of the skin (palms), keratoderma (palms), thickened (toes) or brittle nails (fingers)	*De novo*
	D493-2	Sibling	3	p.R75W/+	Congenital, profound	Thickening of the skin (palms), minor peeling and erythema (palms), brittle nails (fingers)	*De novo*
C209	C209-1*	-	33	p.R184Q/+	Congenital, severe	Thickening and peeling of the skin (soles), keratoderma (soles), callus (soles), thickened nails (fingers), spoon nails (toes)	*De novo*
D40	D40-1*	-	6	p.R184Q/+	Congenital, profound	None	*De novo*

*Probands.

**Figure 2:**
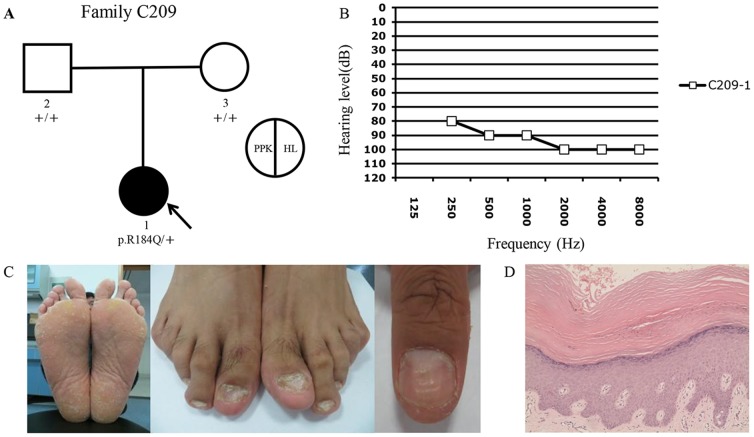
Genotype and phenotype characterization of Fmaily 209. (A) Pedigree and the genotypes of family members. Proband C209-1 with *de novo* p.R184Q mutation of *GJB2* was pointed by the arrow. (B) HL phenotypes of C209-1. (C) PPK phenotypes of C209-1 including the thickening and peeling of the skin, keratoderma, callus (the left panel), spoon nails of the toes (the middle panel) and thickened nails of the fingers (the right panel). (D) Skin biopsy of the left sole showing hyperkeratosis, thickening of the granular layer, acanthosis, and sparse lymphocytes infiltration in the superficial layer of dermis. (Hematoxylin-eosin stain; original magnification ×200.).

All subjects with the p.R75Q (n = 5) and p.R75W (n = 4) mutations had syndromic HL with PPK. C209-1 and D40-1, two subjects with the p.R184Q mutations, had syndromic and non-syndromic HL, respectively. Since the p.R184Q mutation has not been reported to be associated with epidermal abnormalities previously (Table S1), we further characterized the PPK phenotype of subject C209-1 (Figure 2). In addition to a variety of epidermal abnormalities, skin biopsy of his soles confirmed the hyperkeratosis, thickening of the granular layer, acanthosis, and sparse lymphocytes infiltration in the superficial layer of dermis.

### High *de novo* rate of dominant *GJB2* mutations

**Figure 4:**
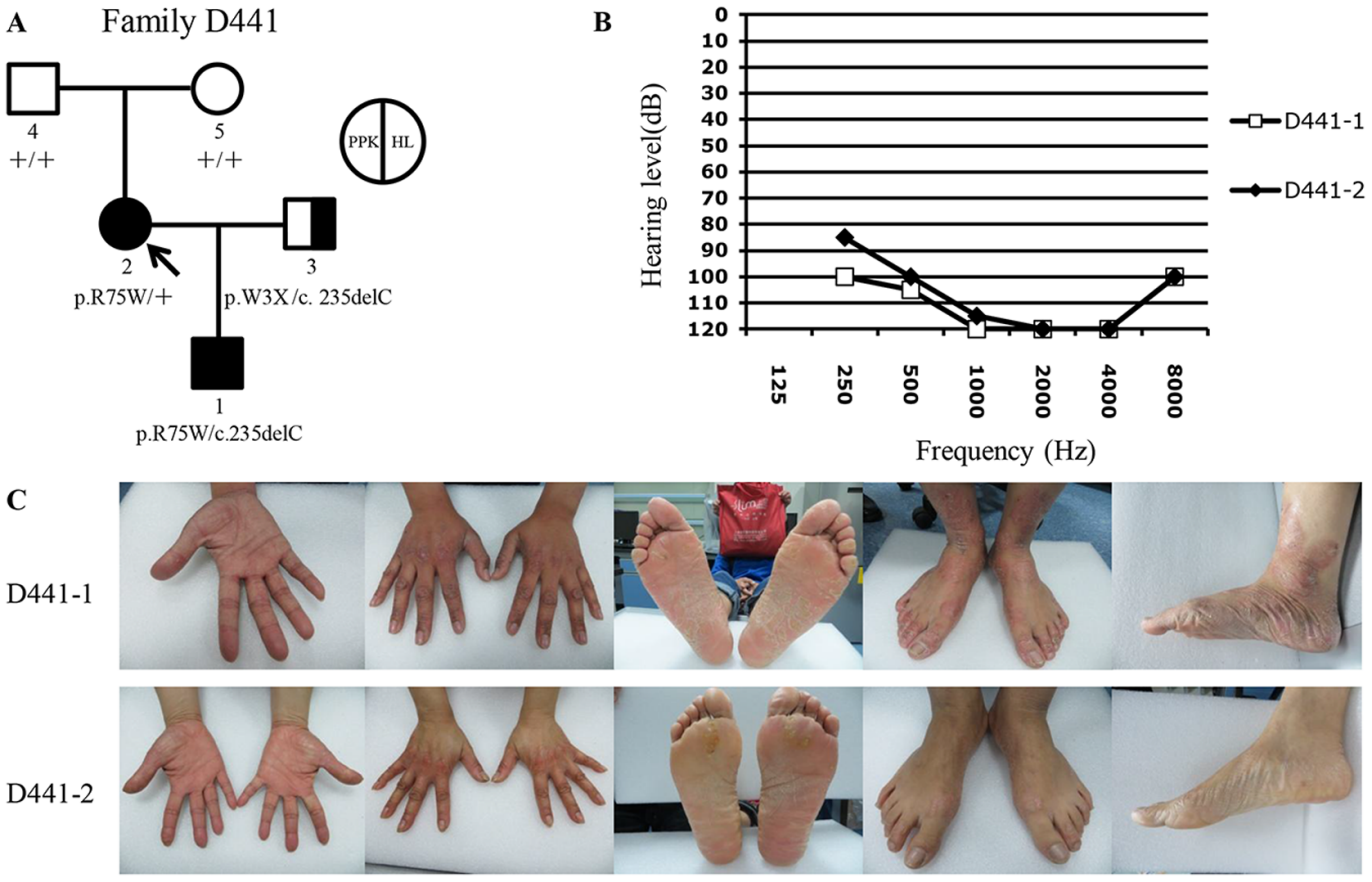
Genotype and phenotype characterization of Family D441. (A) Pedigree and the genotypes of family members. Subject D441-2 with *de novo* p.R75W mutation of *GJB2* was pointed by the arrow. (B) HL phenotypes of D441-1 and D441-2. (C) PPK phenotypes of D441-1 and D441-2. Note that D441-1 had more severe keratoderma (the third panels) and erythema (the second, fourth and fifth panels) than D441-2, and that the PPK phenotype of D441-1 was extended to the wrist (the second panels), the ankle (the fourth and fifth panels) and the back of the hands and feet (the second and fourth panels).

In 6 of the 7 families detected with dominant *GJB2* mutations, the probands (D898-1, D1057-1, D493-1, C209-1 and D40-1) and/or their first-degree relatives (D441-2 and D493-2) had unaffected parents. STR analysis of the trios confirmed the true parenthood (data not shown). Parental genotyping showed that the dominant *GJB2* mutations detected in those 7 subjects were *de novo* (Figure 2A, 4A and Figure S1). One particular pair of siblings, D493-1 and D493-2, both had *de novo* mutations of p.R75W, possibly through parental germline mosaicism.

### Phenotypic modification by additional *GJB2* mutations

The p.R75Q and p.R75W mutations were identified in multiple family members in Family D439 (D439-1, D439-2 and D439-4) and Family D441 (D441-1 and D441-2), respectively. Interestingly, intrafamilial variations of the degree of HL and PPK were observed in both families and the severity of phenotype was apparently modified by the presence of additional *GJB2* mutations.

**Figure 3:**
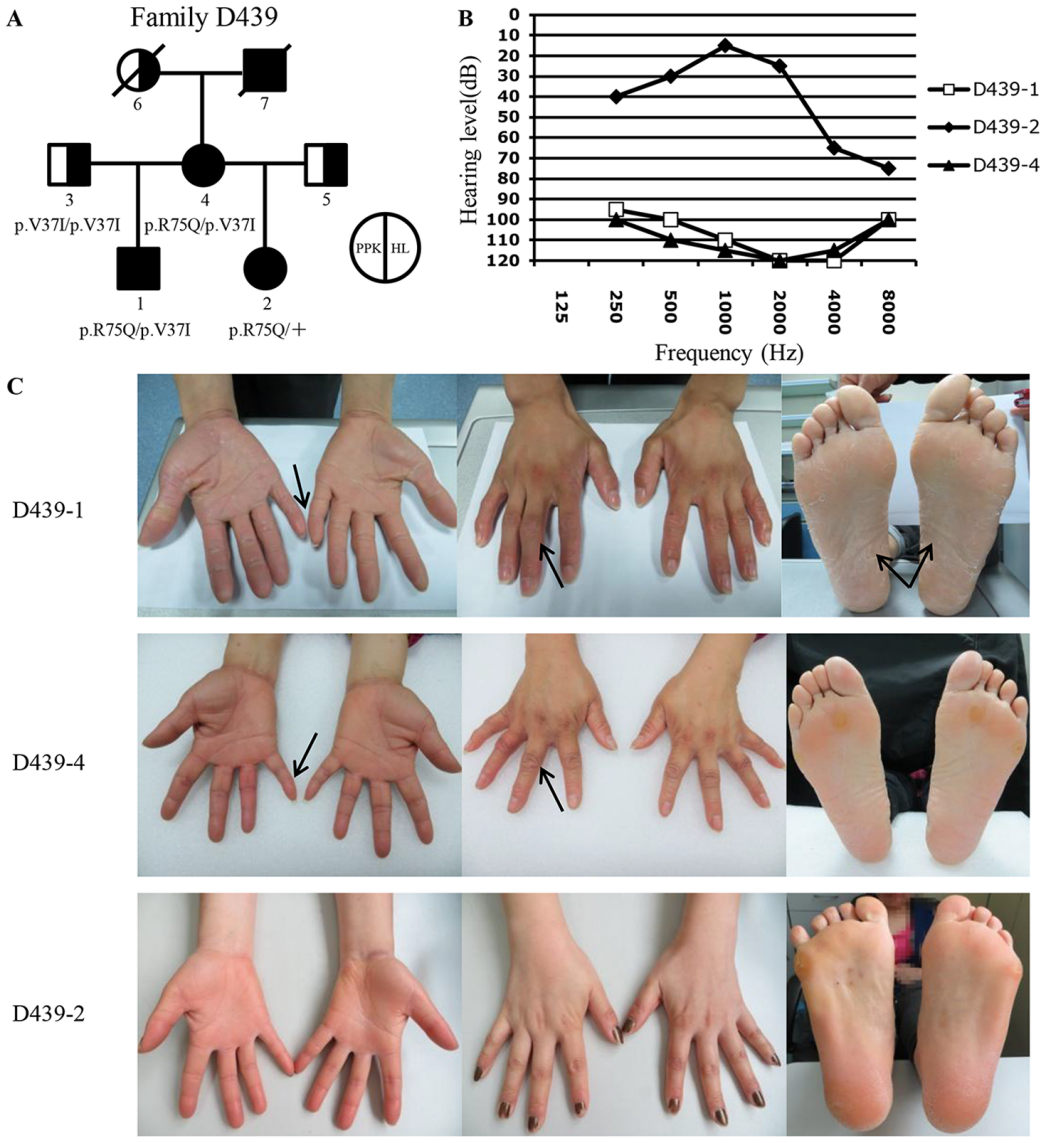
Genotype and phenotype characterization of Fmaily D439. (A) Pedigree and the genotypes of family members. (B) HL phenotypes of D439-1, D439-2 and D439-4. (C) PPK phenotypes of D439-1, D439-2 and D439-4. Note that D439-1 and D439-4 had more severe keratoderma than D439-2 (the left and right panels), and that a number of PPK-associated epidermal abnormalities observed in D439-1 and D439-4 were absent in D439-2, including peeling of the skin (arrows in the right panel), knuckle pad (arrows, the middle panel) and circular keratotic constriction band (arrows, the left panel).

In Family D439, both proband D439-1 and his mother D439-4 had the p.R75Q/p.V37I compound heterozygous mutations of *GJB2*, while the proband's half-sibling D439-2 had the p.R75Q heterozygous mutation only (Figure 3A). Consistent with the genotypes, the HL of both D439-1 and D439-4 was congenital and profound, while that of D439-2 was delayed-onset and mild (Figure 3B). In addition, D439-1 and D439-4 had more severe PPK than D439-2, including symptoms of knuckle pad and circular keratotic constriction band of the fingers that were absent in D439-2.

In Family 441, proband D441-1 had the p.R75W/c.235delC compound heterozygous mutations of *GJB2*, while the proband's mother D441-2 had the p.R75W heterozygous mutation only (Figure 4A). Though the degree of HL was similar (Figure 4B), D441-1 exhibited more severe PPK phenotypes than D441-2, and the affected area was extended to his wrists, ankles and the back of hands and feet (Figure 4C), areas that were typically unaffected in other PPK patients.

## Discussion

In this study, we characterized the spectrum, *de novo* rate and genotype-phenotype correlation of dominant *GJB2* mutations associated with hearing and epidermal disorders in Chinese Hans. Out of 2168 probands with sensorineural HL, 7 (0.32%) was detected with known dominant *GJB2* mutations. Though not as common as its recessive counterpart, dominant mutations in *GJB2* still represent one of the most frequent causes for dominantly inherited HL. To date, more than 20 dominant mutations of *GJB2* has been reported worldwide (The Human Gene Mutation Database, http://www.hgmd.cf.ac.uk). The mutation spectrum revealed by the current study of Chinese Hans, however, evolved only three of them: p.R75Q (n = 3), p.R75W (n = 2) and p.R184Q (n = 2). Considering that all these three mutations have also been reported in sporadic cases with HL in Taiwan or mainland China (Table S1), they are likely among the most common dominant *GJB2* mutations in Chinese Hans.

As summarized in Table S1, the p.R75Q and p.R75W mutations were associated with both non-syndromic and syndromic HL in previous reports, while the p.R184Q mutation was exclusively associated with non-syndromic HL. Interestingly however, in our study all 5 families, 9 patients with the p.R75Q or p.R75W mutations and one patient with the p.R184Q mutation exhibited syndromic HL with PPK (Table 1). Our results suggested that the genotype-phenotype correlation of dominant *GJB2* mutations may be variable among different ethnic groups.

One of the most interesting findings of our study was the very high *de novo* rate of dominant *GJB2* mutations in Chinese Hans, which occurred in 71% (5/7) of the probands and two additional family members with the p.R75Q (n = 2), p.R75W (n = 3) and p.R184Q (n = 2) mutations (Table 1, Figure 2A, 4A and Figure S1). It has been shown that most *de novo* mutations constitute transitions (purine↔purine or pyrimidine↔pyrimidine) rather than transversions (purine↔pyrimidine), and the mutation rate can be elevated at the CpG-rich region [12]. The p.R75Q (CGG>CAG), p.R75W (CGG>TGG) and p.R184Q (CGG>CAG) mutations all fit in with this characteristics of *de novo* mutations, which may partly explain the relatively high frequency of these three dominant *GJB2* mutations in Chinese Hans.

In two families segregated with one dominant and one recessive *GJB2* mutations, family members with both mutations exhibited more severe HL and PPK phenotypes than those with the dominant *GJB2* mutation only (Figure 3 and 4). This intrafamilial variation of the phenotypes leads us to hypothesize that the additional heterozygous recessive mutation of *GJB2* may modify the HL and PPK phenotypes caused by the dominant *GJB2* mutations. Consistent with our observation, the speculated phenotypic modification has also been supported in several other families with dominant *GJB2* mutations [13], [14]. Functional studies are needed to investigate this hypothesized modification effect.

In summary, our study provided a preliminary overview of the spectrum, *de novo* rate and genotype-phenotype correlation of dominant *GJB2* mutations in Chinese Hans. Characterization of these features may result in more accurate genetic testing and counseling of the associated disorders.

## Supporting Information

Figure S1 **Four additional families with** ***de novo*** **mutations of** ***GJB2*** **.** The affected individuals with *de novo* mutations were pointed by the arrows.
(TIF)

Table S1 Genotype-phenotype correlation of the p.R75Q, p.R75W and p.R184Q mutation in previous reports and the current study.
(DOC)
