# Supplementary figures and images for: Characterization of Spectrum, de novo Rate and Genotype-Phenotype Correlation of Dominant GJB2 Mutations in Chinese Hans
Source: PLoS One. 2014 Jun 19;9(6):e100483. doi: 10.1371/journal.pone.0100483 (PMC4063943; doi:10.1371/journal.pone.0100483)

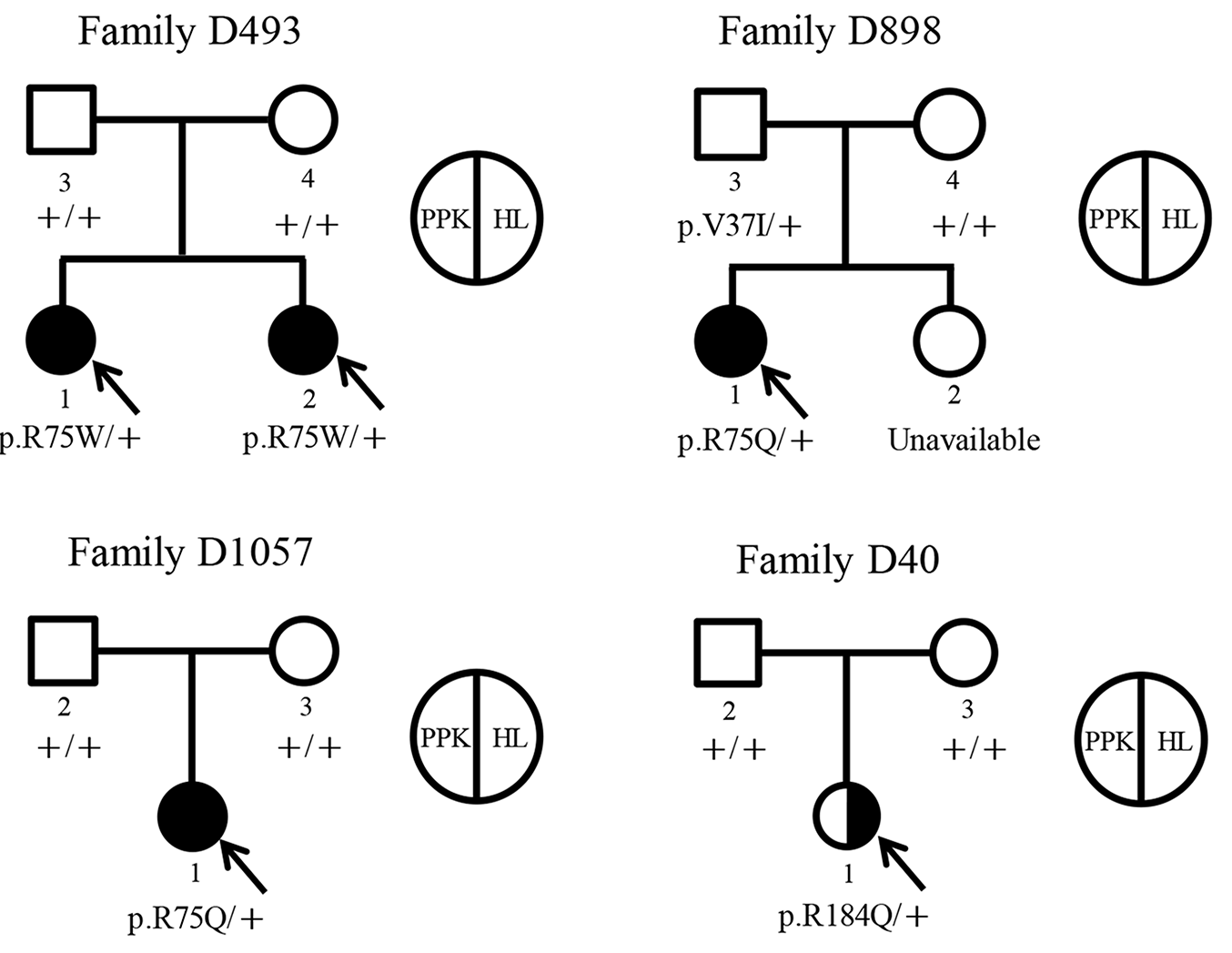

Supplement: Figure S1 — Four additional families with de novo mutations of GJB2 . The affected individuals with de novo mutations were pointed by the arrows. (TIF) [file pone.0100483.s001.tif]
