# Supplementary material for: Characterization of Spectrum, de novo Rate and Genotype-Phenotype Correlation of Dominant GJB2 Mutations in Chinese Hans
Source: PLoS One. 2014 Jun 19;9(6):e100483. doi: 10.1371/journal.pone.0100483 (PMC4063943; doi:10.1371/journal.pone.0100483)
Supplement: Table S1 — Genotype-phenotype correlation of the p.R75Q, p.R75W and p.R184Q mutation in previous reports and the current study. (DOC) [file pone.0100483.s002.doc]

**Supplementary Table S1.** Genotype-phenotype correlation of the p.R75Q, p.R75W and p.R184Q mutation in previous reports and the current study.

| Mutation | Phenotype | Region | Families | Patients | References |
| --- | --- | --- | --- | --- | --- |
| p.R75Q | HI | France  Italy  Germany  Italy  Taiwan  Brazil | 1  1  2  1  1  1 | 2  4  3  2  4  7 |  |
|  | HI, PPK | France  Turkey  Brazil  **China** | 1  1  1  **3** | 3  4  3  **5** | **The present study** |
| p.R75W | HI | Spain  India  Netherland  Austria | 1  2  1  1 | 1  2  1  1 |  |
|  | HI, PPK | Egypt  China  Korea  Germany  **China** | 1  1  1  1  **2** | 2  1  3  1  **4** | **The present study** |
| p.R184Q | HI | Netherland  Iran  Mexico  Slovakia  China  Taiwan  Ghana  **China** | 1  1  1  1  2  2  1  **1** | 2  2  1  1  2  2  1  **1** | **The present study** |
|  | HI, PPK | **China** | **1** | **1** | **The present study** |

Birkenhager R, Lublinghoff N, Prera E, Schild C, Aschendorff A, Arndt S (2010) Autosomal dominant prelingual hearing loss with palmoplantar keratoderma syndrome: Variability in clinical expression from mutations of R75W and R75Q in the GJB2 gene. Am J Med Genet A 152A: 1798-802. doi: 10.1002/ajmg.a.33464

Dalamon V, Beheran A, Diamante F, Pallares N, Diamante V, Elgoyhen AB (2005) Prevalence of GJB2 mutations and the del(GJB6-D13S1830) in Argentinean non-syndromic deaf patients. Hear Res 207: 43-9. doi: 10.1016/j.heares.2005.04.012

de la Luz Arenas-Sordo M, Menendez I, Hernandez-Zamora E, Sirmaci A, Gutierrez-Tinajero D, McGetrick M, Murphy-Ruiz P, Leyva-Juarez X, Huesca-Hernandez F, Dominguez-Aburto J, Tekin M (2012) Unique spectrum of GJB2 mutations in Mexico. Int J Pediatr Otorhinolaryngol 76: 1678-80. doi: 10.1016/j.ijporl.2012.08.005

Feldmann D, Denoyelle F, Blons H, Lyonnet S, Loundon N, Rouillon I, Hadj-Rabia S, Petit C, Couderc R, Garabedian EN, Marlin S (2005) The GJB2 mutation R75Q can cause nonsyndromic hearing loss DFNA3 or hereditary palmoplantar keratoderma with deafness. Am J Med Genet A 137: 225-7. doi: 10.1002/ajmg.a.30765

Hamelmann C, Amedofu GK, Albrecht K, Muntau B, Gelhaus A, Brobby GW, Horstmann RD (2001) Pattern of connexin 26 (GJB2) mutations causing sensorineural hearing impairment in Ghana. Hum Mutat 18: 84-5. doi: 10.1002/humu.1156

Huang S, Yuan Y, Liu J, Han D, Kang D, Zhang X, Dong M, Yan X, Dai P (2011) De novo dominant mutation of GJB2 in two Chinese families with nonsyndromic hearing loss. Int J Pediatr Otorhinolaryngol 75: 1333-6. doi: 10.1016/j.ijporl.2011.07.033

Iossa S, Marciano E, Franze A (2011) GJB2 Gene Mutations in Syndromic Skin Diseases with Sensorineural Hearing Loss. Curr Genomics 12: 475-785. doi: 10.2174/138920211797904098

Janecke AR, Nekahm D, Loffler J, Hirst-Stadlmann A, Muller T, Utermann G (2001) De novo mutation of the connexin 26 gene associated with dominant non-syndromic sensorineural hearing loss. Hum Genet 108: 269-70.

Lee JY, In SI, Kim HJ, Jeong SY, Choung YH, Kim YC (2010) Hereditary palmoplantar keratoderma and deafness resulting from genetic mutation of Connexin 26. J Korean Med Sci 25: 1539-42. doi: 10.3346/jkms.2010.25.10.1539

Mahdieh N, Shirkavand A, Raeisi M, Akbari MT, Tekin M, Zeinali S (2010) Unexpected heterogeneity due to recessive and de novo dominant mutations of GJB2 in an Iranian family with nonsyndromic hearing loss: implication for genetic counseling. Biochem Biophys Res Commun 402: 305-7. doi: 10.1016/j.bbrc.2010.10.021

Mani RS, Ganapathy A, Jalvi R, Srikumari Srisailapathy CR, Malhotra V, Chadha S, Agarwal A, Ramesh A, Rangasayee RR, Anand A (2009) Functional consequences of novel connexin 26 mutations associated with hereditary hearing loss. Eur J Hum Genet 17: 502-9. doi: 10.1038/ejhg.2008.179

Manzoli GN, Abe-Sandes K, Bittles AH, da Silva DS, Fernandes Lda C, Paulon RM, de Castro IC, Padovani CM, Acosta AX (2013) Non-syndromic hearing impairment in a multi-ethnic population of Northeastern Brazil. Int J Pediatr Otorhinolaryngol 77: 1077-82. doi: 10.1016/j.ijporl.2013.04.001

Minarik G, Tretinarova D, Szemes T, Kadasi L (2012) Prevalence of DFNB1 mutations in Slovak patients with non-syndromic hearing loss. Int J Pediatr Otorhinolaryngol 76: 400-3. doi: 10.1016/j.ijporl.2011.12.020

Piazza V, Beltramello M, Menniti M, Colao E, Malatesta P, Argento R, Chiarella G, Gallo LV, Catalano M, Perrotti N, Mammano F, Cassandro E (2005) Functional analysis of R75Q mutation in the gene coding for Connexin 26 identified in a family with nonsyndromic hearing loss. Clin Genet 68: 161-6. doi: 10.1111/j.1399-0004.2005.00468.x

Richard G, White TW, Smith LE, Bailey RA, Compton JG, Paul DL, Bale SJ (1998) Functional defects of Cx26 resulting from a heterozygous missense mutation in a family with dominant deaf-mutism and palmoplantar keratoderma. Hum Genet 103: 393-9.

Uyguner O, Tukel T, Baykal C, Eris H, Emiroglu M, Hafiz G, Ghanbari A, Baserer N, Yuksel-Apak M, Wollnik B (2002) The novel R75Q mutation in the GJB2 gene causes autosomal dominant hearing loss and palmoplantar keratoderma in a Turkish family. Clin Genet 62: 306-9.

Wang YC, Kung CY, Su MC, Su CC, Hsu HM, Tsai CC, Lin CC, Li SY (2002) Mutations of Cx26 gene (GJB2) for prelingual deafness in Taiwan. Eur J Hum Genet 10: 495-8. doi: 10.1038/sj.ejhg.5200838

Weegerink NJ, Pennings RJ, Huygen PL, Hoefsloot LH, Cremers CW, Kunst HP (2011) Phenotypes of two Dutch DFNA3 families with mutations in GJB2. Ann Otol Rhinol Laryngol 120: 191-7.

Wu CC, Lin YH, Lu YC, Chen PJ, Yang WS, Hsu CJ, Chen PL (2013) Application of massively parallel sequencing to genetic diagnosis in multiplex families with idiopathic sensorineural hearing impairment. PLoS One 8: e57369. doi: 10.1371/journal.pone.0057369

Yang JJ, Huang SH, Chou KH, Liao PJ, Su CC, Li SY (2007) Identification of mutations in members of the connexin gene family as a cause of nonsyndromic deafness in Taiwan. Audiol Neurootol 12: 198-208. doi: 10.1159/000099024

Yuan Y, Huang D, Yu F, Zhu X, Kang D, Yuan H, Han D, Dai P (2009) A de novo GJB2 (connexin 26) mutation, R75W, in a Chinese pedigree with hearing loss and palmoplantar keratoderma. Am J Med Genet A 149A: 689-92. doi: 10.1002/ajmg.a.32461
